# Resveratrol Protects against Restraint Stress Effects on Stomach and Spleen in Adult Male Mice

**DOI:** 10.3390/ani9100736

**Published:** 2019-09-27

**Authors:** Wael Ennab, Sheeraz Mustafa, Quanwei Wei, Zengpeng Lv, Ngekure M.X. Kavita, Saif Ullah, Fangxiong Shi

**Affiliations:** College of Animal Science and Technology, Nanjing Agricultural University, Nanjing 210095, China; Wael91.Ennab@gmail.com (W.E.); sheerazmustafa786@gmail.com (S.M.); weiquanwei@njau.edu.cn (Q.W.); lvzengpeng@njau.edu.cn (Z.L.); balochpz30@gmail.com (S.U.)

**Keywords:** resveratrol, restraint stress, apoptosis-inducing factor, parietal cells, DAB intensity, splenomegaly

## Abstract

**Simple Summary:**

The current project was designed to examine the effects of restraint stress on body weights, food and water consumption, and pathophysiology of the stomach and spleen in adult mice, and potential roles of the phenolic compound resveratrol during treatment. We found that restraint stress (which is known to be a mixture of psychologic and physical stress) caused a reduction in body weight, food and water consumption, and morphologic changes in the stomach and spleen, which could lead to gastritis or ulcers and splenomegaly, while treatment with resveratrol prevented the negative alterations to the stomach and spleen.

**Abstract:**

The objectives were to investigate whether restraint stress (which is known as a mixture of psychologic and physical stress) exerts negative effects on the stomach and spleen, and whether the phenolic compound resveratrol (RES) exerts any protective roles. Fifty adult male mice were divided into five groups, with 10 mice per group as follows: control (C), restraint stress (RS), RS with vehicle (RS + V), RS with 2 mg/kg of resveratrol (RS + 2 mg RES), and RS with 20 mg/kg of resveratrol (RS + 20 mg RES). Mice were restrained in conical centrifuge tubes for 4 h daily to establish the RS model. RS + 2 mg RES, RS + 20 mg RES, and RS + V groups were given an oral dose of resveratrol or vehicle for 15 consecutive days, while the control group was not exposed to restraint stress. Herein, we showed that restraint stress decreased body weight and food and water consumption in stressed groups RS and RS + V compared to controls, while the groups treated with resveratrol showed improvements. Moreover, restraint stress caused acute damage to the morphology of gastric cells and reduced the quantitative distribution of parietal cells along with their decreased size and diameter, pointing to gastritis or ulcer. Furthermore, the antibody against the apoptosis-inducing factor (AIF) was highly attached in the RS groups. Splenic size, weight, and length were also greatly augmented in the stressed groups compared to the controls, while these phenomena were not observed in the RS + 2 mg RES group. Our findings proved significant ameliorating effects of resveratrol against restraint stress in adult male mice.

## 1. Introduction

Stress is an inevitable phenomenon in this modern world. Stressful situations can lead to many physiological and psychological alterations. Disturbance of homeostasis is called stress, and homeostasis challenged by a stimulus is termed as a stressor, such as an environmental condition [1]. Generally, there are three types of stressors, physical, psychological, and metabolic. In experimental models for biomedical research, stressors are often used in a mixed type such as restraint stress, where movement is limited to a restricted area, and the individual animal or human being is isolated from his or her group [2,3]. Under stress, the energy requirement of the body increases, resulting in increased production of free radicals. In addition, the body loses its ability to defend against stress [4]. Stress response research has enabled us to unravel the connection between stress and certain systematic dysfunctions that can lead to multiple pathophysiologic disorders, including inflammatory disorders or chronic inflammation [5]. Serious stress can affect metabolism and growth, causing different acute disorders [6], such as weakening the digestive processes, immunity, and endocrine responses [7]. Stress may also lead to gastrointestinal disorders [8,9,10].

The stomach is the most important organ in the body for the food digestion process. The parietal cells in the stomach occupy a large area due to their large size and their extension over the gastric glands [11], and the potential of the stomach to release its secretions is known to be linked almost linearly to parietal cell numbers [12]. In comparison, chronic inflammation leads to mucosal atrophy, with a reduced parietal cell number [13]. Furthermore, when parietal cells are lost (which may occur with chronic inflammation), the chief cells cannot mature [14,15].

The spleen serves as a hematopoietic and secondary lymphoid organ in mice. It is the largest immune organ in the body and is also necessary for homeostasis and iron recycling of red blood cells [16]. Many gastrointestinal diseases—such as coeliac disease, chronic atrophic gastritis, and inflammatory intestinal illness—may trigger splenic disorders (e.g., splenomegaly) as secondary symptoms because they possess similar pathophysiology [17].

Research strategies against stress are exciting and controversial topics in animal sciences. Compounds derived from herbs and plants (e.g., resveratrol) have been proven to be effective as herbal remedies against stress [18]. Resveratrol (*3,5,4′-trihydroxy*-*trans*-stilbene) is a polyphenol found in grape skin and red wine, and it has received attention due to its reported multiple health benefits [19]. A study has revealed that resveratrol treatment is a promising strategy for therapy and prophylaxis against intestinal inflammation [20]. However, potential roles of resveratrol against stress effects on the stomach and spleen in animals are not yet fully elucidated.

Apoptosis-inducing factor (AIF) is a flavoprotein involved in initiating a caspase-independent pathway of apoptosis by causing DNA fragmentation and chromatin condensation [21], and AIF acts as an NADH oxidase and regulates the permeability of the mitochondrial membrane upon apoptosis [22]. A reduction in AIF caused acute mitochondrial dysfunction, neuro-degeneration, and occasional muscle atrophy in model organisms as well as in humans [23]. It generally occurs in mitochondria but translocates further to the nucleus when apoptosis is induced [24]. Apoptosis occurs in response to noxious agents, immune reactions, and in cells damaged by disease as a defense mechanism [25]. It was, therefore, interesting to examine AIF expression in the stomach of mice during restraint stress.

It is well known that restraint stress can cause damages to the cellular structure of various organs in the living body. A recent study shows that resveratrol can alleviate the damage caused by restraint stress [26]. We, therefore, designed the present study to clarify the effects of restraint stress on gastric and splenic cells of adult male mice and to investigate whether resveratrol can reverse these untoward effects.

## 2. Materials and Methods

### 2.1. Animals

Young adult male laboratory mice (*Mus musculus*, 25–30 g, 30 days of age) of the Swiss ICR (Institute for Cancer Research) were purchased from the Qinglongshan Laboratory Animal Company (Nanjing, China), and were kept in an animal facility under a controlled environment consisting of a 12 h light/12 h dark cycle, humidity of 60–70%, and room temperature at 22–23 °C. Fifty mice were kept in 5 cages with a density of 10 mice per cage (45 × 30 × 20 cm^3^, length × width × height). For the caging system, we provided a low-stress environment (improved air quality, single-pass air flow) and allergy protection (closed system, isolation, and containment). Cages were also cleaned every 2 days and bedding changed, any dirty cage was removed from the rack at the time to avoid excessive ammonia and carbon dioxide levels on the table and cleaned with disinfectant, and after cleaning the cages, forceps were used to transfer the mice. When applicable, the cage was closed gently and brought back to the rack. Dirty wire grids were replaced for cleaning once a week. Mice cages were placed close to the room walls for quality enrichment of mouse husbandry. The mice were fed using standard balanced rodent pellets (Jiangsu Synergistic Pharmaceutical Bioengineering Co., Limited, Nanjing, China), and clean, fresh drinking water was made available *ad libitum* with daily checking for water bottles and refilled for proper operation and cleanliness. Animals were adapted to handling for 7 days prior to the beginning of the experiment. The experimental protocols involving mice were approved in accordance with the Guide for Care and Use of Laboratory Animals prepared by the Institutional Animal Care and Use Committee of Nanjing Agricultural University (permit number SYXK (Su) 2011-0036), Nanjing, China. All procedures for animal handling were conducted under protocols approved by the Animal Welfare Committee of Nanjing Agricultural University, China.

### 2.2. Restraint Stress Protocol

According to previously reported methods [27], mice were physically restrained in a 50 mL conical centrifuge tube with a diameter of 6 cm. Eight holes 0.4 cm in diameter were made for ventilation. Individual mice were restrained in the tubes without food or water for 4 h a day for 15 consecutive days. Control mice were left in their usual cages for the same duration without food or water [28].

### 2.3. Experimental Design

Resveratrol (*3,5,4′-trihydoxy-trans-stilbene*) (RES; Sigma Chemical Co., St Louis, MO, USA) was dissolved in 10 mL of 5% vehicle (V, carboxymethylcellulose). Fifty adult male mice were divided into 5 groups (10 mice per group), with 1 control group not exposed to restraint stress, and 4 experimental groups exposed to restraint stress (RS) with or without treatments. Groups were as follows: C-group mice were not exposed to restraint stress, RS-group mice were exposed to restraint stress without treatment, RS + V group mice were exposed to similar stress conditions and received an oral dose via gavage of 0.1 mL of V after applied restraint stress once per day, and the RS + 2 mg RES and RS + 20 mg RES groups were exposed to the same restraint stress conditions and received an oral dose via gavage of either 0.1 mL of 2 mg/kg resveratrol or 20 mg/kg of resveratrol after application of restraint stress once a day, respectively, throughout the entire experimental period. Body weight, and food and water consumption were measured daily after exposing the mice to the restraint stress. All animals were sacrificed using cervical dislocation under CO_2_ anesthesia after 15 days of restraint stress. Spleen weight was measured immediately after sacrifice. Fresh splenic and gastric samples were collected and fixed in 4% paraformaldehyde (PFA) for histologic and immunohistochemical examination (Figure S1).

### 2.4. Body Weight and Food and Water Consumption Indexes

Body weight and food and water consumption for experimental groups were measured daily for 15 days after exposing the mice to stress. Water consumption index (mL/g) = total water consumed per day/total body weight, and the food consumption index (g/g) = total food consumed per day/total body weight.

### 2.5. Histologic Analysis

After fixing the stomach and spleen in 4% PFA for 24–48 h, tissues were dehydrated through a graded series of ethanol, cleared with xylene, and embedded in paraffin wax. Sections were cut at 5 μm, stained with routine H&E, and we observed any histopathologic changes microscopically (Nikon ECLIPS 80i and OLYMPUS BX51, Tokyo, Japan).

### 2.6. Measurements of Parietal Cell Diameter

For this method, we used ImageJ software from the National Institutes of Health (ImageJ, version 1.50, NIH, Bethesda, MD, USA, http://imagej.nih.gov/ij/). Cell diameter was calculated based on area measurements after inserting the images into the software, and lines were drawn randomly around 100 parietal cells and divided into 5 groups. Each group contained 20 parietal cells.

### 2.7. Immunohistochemistry (IHC)

We performed immunohistochemical staining using monoclonal antibodies to AIF (Santa Cruz Biotechnology, Santa Cruz, CA, USA; 1:100 dilutions) with the SABC (streptavidin-biotin complex) method in order to quantify gastric cell apoptosis. A few sections were picked randomly from the serial sections and mounted on slides coated with APES (3-aminopropyl-triethoxysilane), which we dried for 24 h at 37 °C. Several slides were randomly selected, and we deparaffinized and rehydrated the slides in a series of graded dimethylbenzene and ethylalcohol, and epitope exposure was promoted in boiling water with 20% sodium citrate for 4 min. We then quenched the endogenous peroxidases with a methanol buffer containing 2.5% H_2_O_2_ for 1 h. Next, nonspecific staining was blocked with 5% bovine serum albumin (BSA) for 1.5 h at room temperature, and then slides were incubated with the primary antibody (diluted 1:100 in PBS containing 1% BSA) overnight at 4 °C. The immune reactivity of a specific protein was visualized with 0.05% 3, 3′-diaminobenzidine tetrachloride (DAB; D8001; Sigma Chemical Co., St Louis, MO, USA) in 10 m*M* PBS containing 0.01% (*v/v*) H_2_O_2_ for 2 min, and was counterstained with hematoxylin. The negative control (NC) was normal rabbit serum (NRS) instead of the primary antibody. The immune reactivity of target proteins was detected using rabbit IgG or mouse IgG-SABC kits (No. SA2002/SA2001; Boster Biological Technology, Wuhan, China), and visualized with 0.05% DAB in 10 mmol/L PBS containing 0.01% H_2_O_2_ for 60 s. Finally, the images were captured using a virtual light microscope (Model BX51TF, Olympus Corporation, Tokyo, Japan) after counterstaining with hematoxylin. Three independent, blinded observers were asked to examine the photomicrographs for relative levels of immunostaining, and this was repeated at least 5 times. The methods were according to previous studies performed in our laboratory [24,25,26,27,28,29,30,31,32,33,34], as follows: −, no staining detected; +, weak; ++, moderate; +++, strong staining.

### 2.8. IHC Quantification through Digital Image Analysis

The measurement of DAB color intensity using the image pixels was performed according to a previously published method [35] and in accordance with our previous laboratory study [36]. Briefly, the DAB-and hematoxylin-stained IHC digital images captured at 400× magnification were used for analysis through ImageJ software that contained a compatible plug-in of the IHC profiler. We took 3 separate images for DAB, hematoxylin, and the threshold selection of the antibody areas using the threshold function of ImageJ. (NIH, Bethesda, MD, USA). A complementary output was produced by automated integrating deconvolution and by a simple choice of the program menu. The histogram profiling and scoring could be viewed in seconds. 

The digital image analysis requires standards of color pixel intensity values that range from 0 to 255, where 0 describes the darkest shade of color and 255 depicts the lightest shade of color. In the current study, the expression of AIF in parietal cells and the histogram profile reading were less than the number of color pixels (<66%), such that score computation was performed through a given formula:

(1) $$\mathrm{Score}=\frac{\left(\mathrm{Number}\;\mathrm{of}\;\mathrm{pixels}\;\mathrm{in}\;a\;\mathrm{zone}\right)\times \left(\mathrm{Score}\;\mathrm{of}\;\mathrm{the}\;\mathrm{zone}\right)}{\mathrm{Total}\;\mathrm{number}\;\mathrm{of}\;\mathrm{pixels}\;\mathrm{in}\;\mathrm{the}\;\mathrm{image}}$$

We independently analyzed a total of 60 images with the aid of 2 histopathology experts, and the results were compared via 1-way ANOVA.

### 2.9. Spleen Size, Length, and Weight

A camera (Nikoncoolpix p900) was used to photograph the spleen. The scale bar was included directly by using a ruler. We determined the mean of the spleen using ImageJ software from the National Institutes of Health (ImageJ, version 1.50, NIH, Bethesda, MD, USA, http://imagej.nih.gov/ij/).

### 2.10. Statistical Analysis

We used 1-way analysis of variance (1-way ANOVA) followed by Tukey’s post-hoc test to analyze all data. *p* values less than 0.05 were considered statistically significant [37]. 

## 3. Results

The stressed mice showed general weakness, lacked movement, and cleaned themselves instead of consuming water and food after release from the stress tubes.

### 3.1. Effects of Restraint Stress on Body Weight and Food and Water Consumption

**Figure 1:**
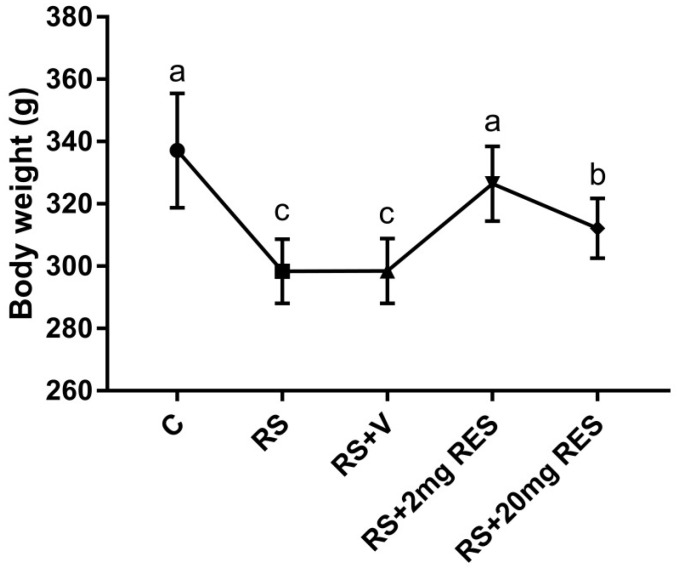
Effects of resveratrol on the body weights of non-stressed mice (C), restraint-stressed mice (RS), restraint-stressed mice treated with vehicle (RS + V), restraint-stressed mice treated with 2 mg/kg body weight of resveratrol (RS + 2 mg RES), and restraint-stressed mice treated with 20 mg/kg body weight of resveratrol (RS + 20 mg RES). Statistical differences were determined by 1-way ANOVA followed by Tukey’s multiple-comparison test. Different superscript letters represent significant differences among groups (*p* < 0.05).

**Figure 2:**
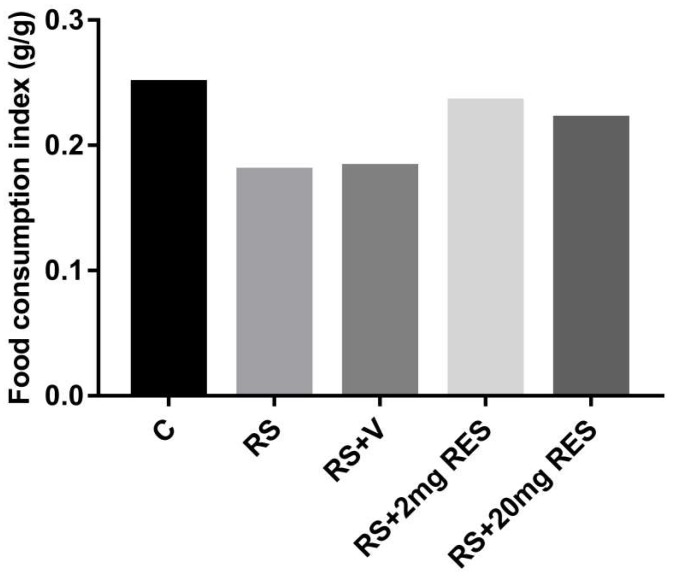
Effects of resveratrol on food consumption index of non-stressed mice (C), restraint-stressed mice (RS), restraint-stressed mice treated with vehicle (RS + V), restraint-stressed mice treated with 2 mg/kg body weight of resveratrol (RS + 2 mg RES), and restraint-stressed mice treated with 20 mg/kg body weight of resveratrol (RS + 20 mg RES). The columns represent the average food consumption index during the 15-day treatment. Food consumption index (g/g) = total food consumed per day/total body weight.

**Figure 3:**
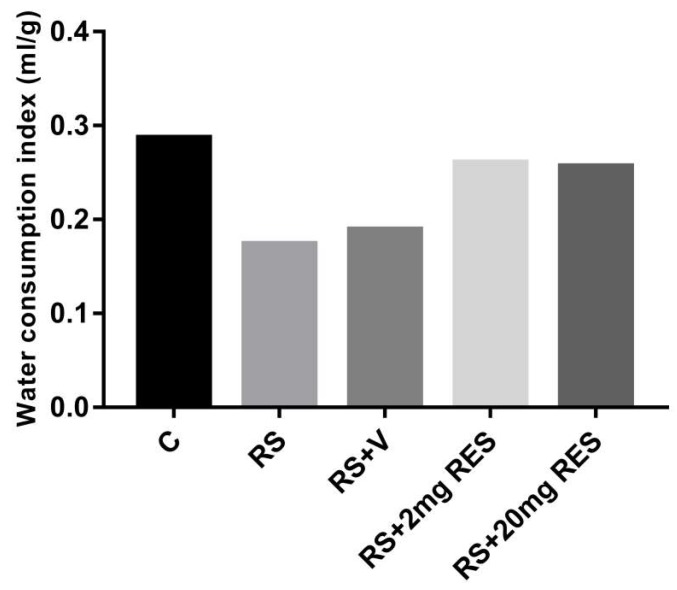
Effects of resveratrol on water consumption index of non-stressed mice (C), restraint-stressed mice (RS), restraint-stressed mice with vehicle (RS + V), restraint-stressed mice treated with 2 mg/kg body weight of resveratrol (RS + 2 mg RES), and restraint-stressed mice treated with 20 mg/kg body weight of resveratrol (RS + 20 mg RES). The columns represent the average water consumption index during the 15-day treatment. Water consumption index (mL/g) = total water consumed per day/total body weight.

The healthy mice of the control group showed no change and a normal increase in body weight as compared to the stressed groups with or without treatment. In addition, the group receiving RS + 2 mg RES showed increased body weight daily during the entire experimental period, while mice in the RS + 20 mg RES, RS, and RS + V groups showed a significant reduction in body weight (*p* < 0.05) (Figure 1). The food consumption in the control and RS + 2 mg RES groups was higher compared to stressed groups RS, RS + V, and RS + 20 mg RES, which showed a reduction of food consumption (Figure 2). Furthermore, the water consumption in control mice and the RS + 2 mg RES group was higher compared to the stressed groups RS, RS + V, and RS + 20 mg RES, which showed a reduction of water consumption during the experimental period (Figure 3).

### 3.2. Effects of Resveratrol on Gastric Histology after Restraint Stress

**Figure 4:**
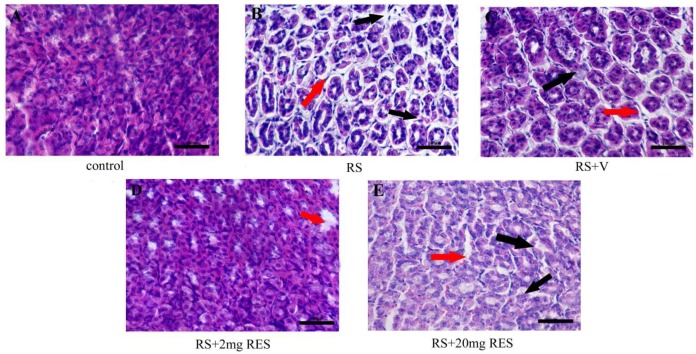
Histopathologic analysis of parietal cells of non-stressed mice (Control), restraint-stressed mice (RS), restraint-stressed mice with vehicle (RS + V), restraint-stressed mice treated with 2 mg/kg body weight of resveratrol (RS + 2 mg RES), and restraint-stressed mice treated with 20 mg/kg body weight of resveratrol (RS + 20 mg RES) using H&E staining. Red arrowheads point to an acute loss of parietal cells. Black arrowheads point to severe damage to the general structure of the gastric mucosa. Scale bar is 100 µm.

We observed that the stressed groups RS, RS + V, and RS + 20 mg RES showed a clearly acute loss of parietal cells with significant injury compared to the control and RS + 2 mg RES groups. Furthermore, we noted that parietal cell size decreased in the RS and RS + V groups compared to controls, RS + 2 mg RES, and RS + 20 mg RES. In addition, we observed large spacing between the cells of the RS and RS + V groups compared to the controls and the RS + 2 mg RES group, which appeared normal. This wide spacing was also seen in the RS + 20 mg RES group, but with more improvement compared to RS and RS + V. The control group showed no histologic differences, with a normal arrangement and characteristic “fried-egg” appearance; and with a basophilic, centrally located nucleus, and a rather eosinophilic cytoplasm. The structures were also normal in the treatment group (RS + 2 mg RES), which protected and improved the general structure of the parietal cells in the stomach compared to the stressed groups RS, RS + V, and RS + 2 0mg RES. It should be noted that the RS + 20 mg RES group achieved some improvements in the general structure of the parietal cells compared to stress groups RS and RS + V (Figure 4).

### 3.3. Effects of Restraint Stress on Parietal Cell Diameter

**Figure 5:**
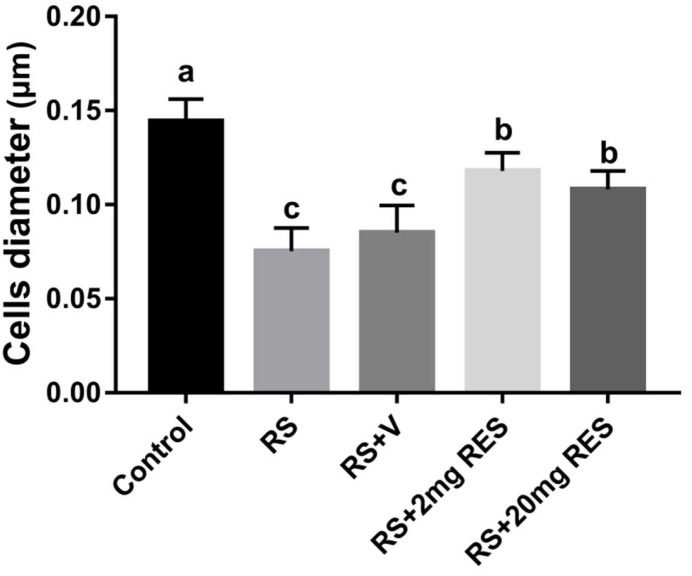
Effects of resveratrol on the parietal cell diameter of non-stressed mice (Control), restraint-stressed mice (RS), restraint-stressed mice with vehicle (RS + V), restraint-stressed mice treated with 2 mg/kg body weight of resveratrol (RS + 2 mg RES), and restraint-stressed mice treated with 20 mg/kg body weight of resveratrol (RS + 20 mg RES). Statistical differences were determined by 1-way ANOVA followed by Tukey’s multiple-comparison test. Different superscript letters represent significant differences among groups (*p* < 0.05).

Along with morphologic variations, we observed that the stressed groups RS and RS + V had a significant reduction in parietal cell size compared with the controls (*p* < 0.05). In contrast, the RS + 2 mg RES, and RS + 20 mg RES groups showed significant improvements in the size of the parietal cell compared to the stressed groups RS and RS + V (*p* < 0.05) (Figure 5).

### 3.4. Immunohistochemical Staining of Parietal Cells of the Mouse Stomach

**Figure 6:**
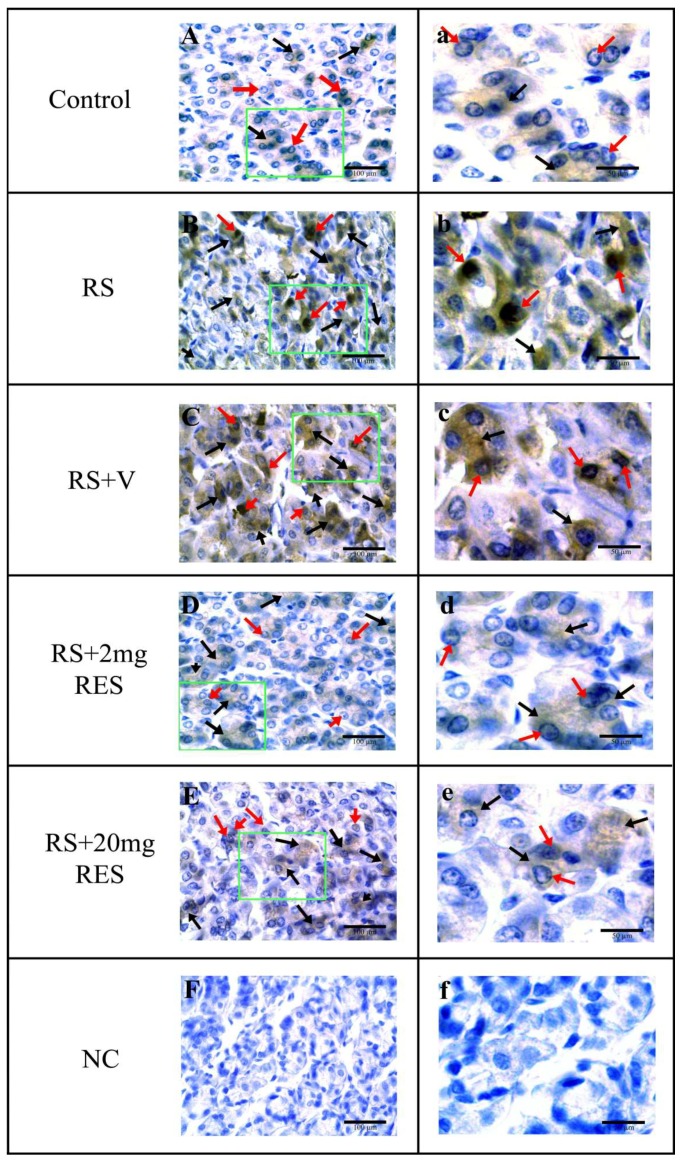
Immunohistochemical observations of apoptosis-inducing factor (AIF) in parietal cells of non-stressed mice (Control), restraint-stressed mice (RS), restraint-stressed mice with vehicle (RS + V), restraint-stressed mice treated with 2 mg/kg body weight of resveratrol (RS + 2 mg RE), restraint-stressed mice treated with 20 mg/kg body weight of resveratrol (RS + 20 mg RES), and the negative control (NC). Red arrows point to the nucleus and black arrows point to the cytoplasm. Scale bar is 100 µm (**A**–**F**) and 50 µm (**a**–**f**).

**Figure 7:**
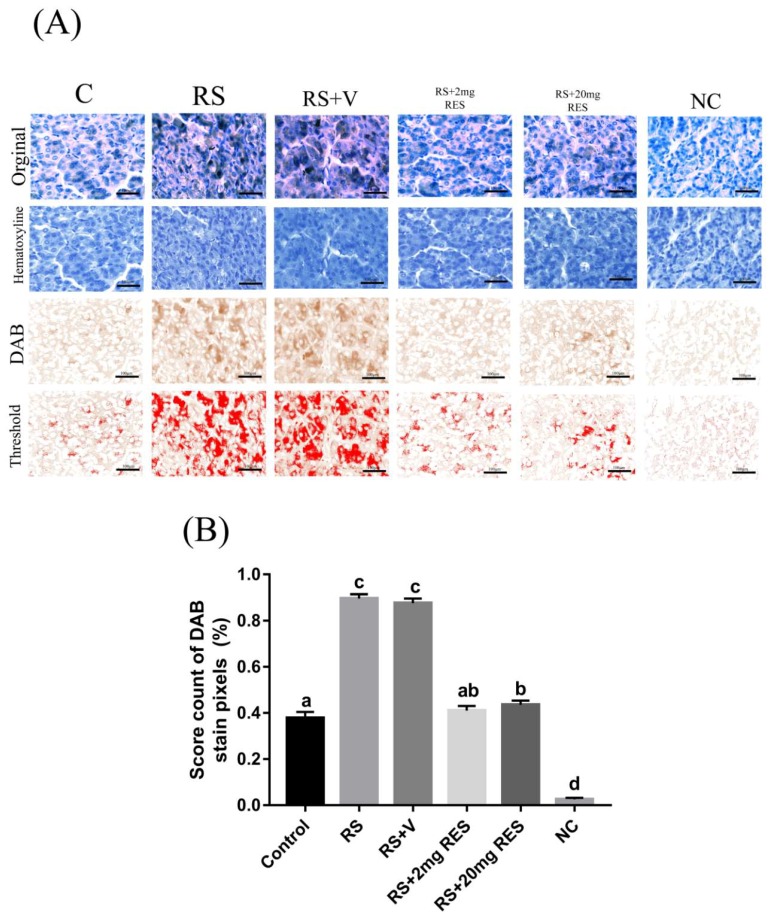
Representative digital images of the histogram profile showing 3, 3′-diaminobenzidine (DAB) brown staining and color pixel intensity for AIF in the parietal cells of the mouse stomach. The histogram profile corresponds to the pixel intensity value vs. the corresponding number of counts of pixel intensity. From top to bottom rows, panels show the digital image masks stained with hematoxylin, DAB, and threshold, respectively. The threshold function of ImageJ was used to place red spots on the DAB stains by setting different threshold levels, with the lower threshold at 0 and upper threshold at 110. (**A**) AIF is expressed in a limited quantity at the tight junction in parietal cells. Each bar in the given graph (**B**) represents the mean ± SEM (n = 12). Statistical differences were determined by 1-way ANOVA followed by Tukey’s multiple-comparison test. Different superscript letters represent significant differences among groups (*p* < 0.05).

Parietal cell staining showed that the stressed groups RS and RS + V exhibited a strong expression of AIF, and although the RS + 20 mg RES group manifested an expression of AIF, it was less than that of the RS and RS + V groups. Similarly, this expression was not elevated compared to the RS + 2 mg RES group. In contrast, the control and RS + 2 mg RES groups showed normal staining expression. No specific expression was found in the negative control group (NC) (Figure 6, Table 1, and Figure 7A,B).

### 3.5. Effects of Restraint Stress on Spleen Size, Length, and Weight

**Figure 8:**
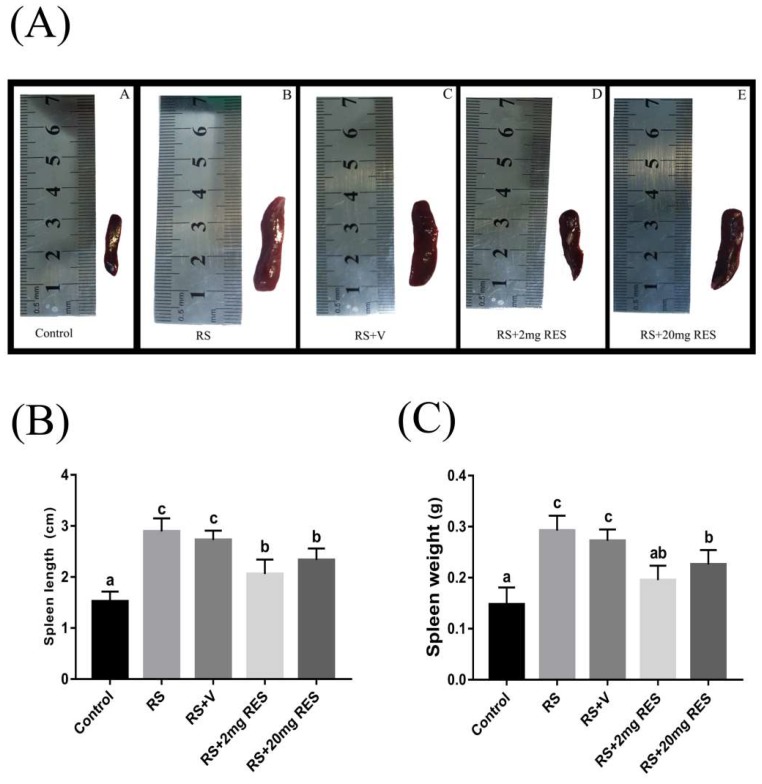
Resveratrol and restraint stress effects on spleen size (**A**), length (**B**), and weight (**C**) of non-stressed mice (Control), restraint-stressed mice (RS), restraint-stressed mice with vehicle (RS + V), and restraint-stressed mice treated with 2 mg/kg body weight resveratrol (RS + 2 mg RES), and restraint-stressed mice treated with 20 mg/kg body weight of resveratrol (RS + 20 mg RES). Each bar in the given graphs represents the mean ± SEM (n = 10). Statistical differences were determined by 1-way ANOVA followed by Tukey’s multiple comparison test. Different superscript letters represent significant differences among groups (*p* < 0.05).

We observed that the spleen of stressed groups RS and RS + V was enlarged compared to the control and RS + 2 mg RES groups, which showed normal splenic size. The RS + 20 mg RES group showed some improvements in spleen size compared to the stressed groups RS and RS + V, which were abnormally enlarged. The experimental data also showed significant differences in spleen length and weight between the control and all stressed group (RS, RS + V, RS + 2 mg RES, and RS + 20 mg RES) (*p* < 0.05), while the RS + 2 mg RES and RS + 20 mg RES groups showed significant improvements compared to the stressed groups RS and RS + V (*p* < 0.05) (Figure 8A–C).

### 3.6. Effects of Resveratrol on Histologic Alterations in the Spleen Due to Restraint Stress

**Figure 9:**
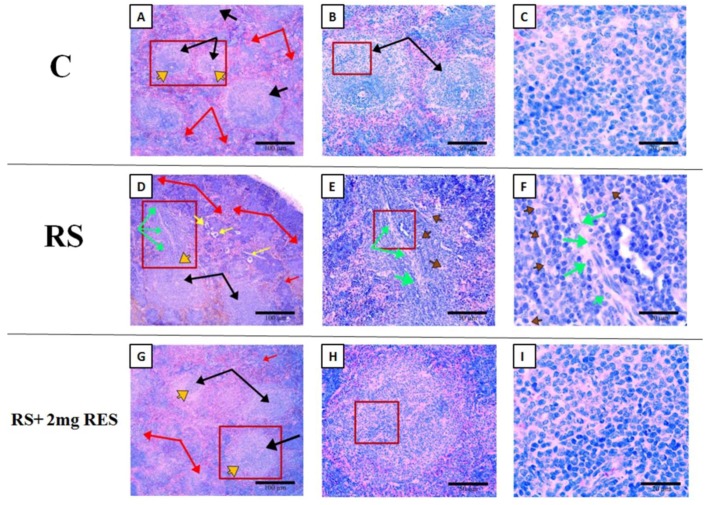
Histopathologic analysis of H&E staining of the splenic structures of non-stressed control mice (C), restraint-stressed mice (RS), and restraint-stressed mice treated with 2 mg/kg body weight of resveratrol (RS + 2 mg RES). Scale bar is 100 µm (**A**,**D**,**G**), 50 µm (**B**,**E**,**H**), and 20 µm (**C**,**F**,**I**). (**A**,**D**,**G**) Black arrows point to the white pulp. (**A**,**D**,**G**) Red arrows point to the red pulp. There is disruption of the normal splenic architecture by a diffuse infiltrate predominantly expanding the white pulp (**D**, green arrows), loss of typical structure of the germinal center (**G**,**C**) (**D**, orange arrows), proliferation of large atypical lymphoid cells with bizarre nuclei (**E** and **F**, brown arrows), and prominent eosinophilic nucleoli surrounded by abundant cytoplasm (**E** and **F**, green arrows). The red pulp was also enlarged and increased (**D**, red arrows), containing extramedullary hematopoiesis and prominent megakaryocytes (**D**, yellow arrows) in the RS group relative to the controls (**A**–**C**) and RS + 2 mg RES (**G**–**I**) groups, which show normal histology in the white and red pulp of the spleen.

According to the above results regarding spleen size, weight, and length, we selected three representative groups for further analysis of splenic histology as follows: control, RS, and RS + 2 mg RES. Our histologic observations showed differences in splenic histopathology among control, RS, and RS + 2 mg RES groups. There was disruption of the normal splenic cell structure by a diffuse infiltrate that predominantly expanded the white pulp in the RS group, while the control and RS + 2 mg RES groups showed normal structures and a healthy white pulp. Furthermore, the white pulp of the RS group demonstrated the proliferation of large atypical lymphoid cells, with bizarre nuclei and prominent eosinophilic nucleoli surrounded by abundant cytoplasm; while in the control and RS + 2 mg RES groups, the white pulp was normal. In addition, the splenic red pulp was also enlarged and showed increased extramedullary hematopoiesis (EMH), and contained prominent megakaryocytes in the RS group—which could lead to splenomegaly. These pathologic changes did not appear in the control and RS + 2 mg RES groups (Figure 9).

## 4. Discussion

Unexpected short-and long-term stimuli can trigger a stress response in an animal’s body that forces the internal environment to resist or adapt to these adverse reactions. In fact, stress affects every organ in the body [38]. Resveratrol is a stilbenoid (a type of natural phenolic compounds) secreted by plants that can combat stressful damage [39,40]. The present study dealt with effects caused by restraint stress and the role of resveratrol in preventing harmful effects.

Food and water consumption manifests a sensitive response to restraint stress because of longer-lasting anorexia that can influence growth and health, and also because endpoints can be quantified with minimal distress to the animal [41]. Recent research has proven that restraint stress can impair growth performance and food and water consumption [42,43]. Similarly, in our study, restraint stress showed significant negative effects on these same parameters. However, resveratrol treatment decreased the negative effects of restraint stress in terms of growth performance and food and water consumption.

In the present study, we found that restraint stress caused gastric mucosal injury, damaging the normal morphology of parietal cells. A previous study showed that different kinds of stress (physical and psychological) could cause gastric mucosal injury (inducing apoptosis [44]), and gastritis [45]. Resveratrol has been known to prevent the changes and damage caused by restraint stress [46,47,48]. Moreover, it was found that antioxidants such as resveratrol (in low oral doses) protected the structure of colonic mucosa after intestinal inflammation [49,50]. In other models, histologic changes in the pancreas were ameliorated by resveratrol [51]. Such natural compounds with antioxidant properties are also used in popular medicine for gastric ulcers [52]. Our data showed that the treatment of resveratrol healed gastric mucosal injury caused by restraint stress. In general, high doses of any medicine can cause an adverse effect. We, therefore, applied low (2 mg/kg) and high doses (20 mg/kg) of resveratrol, and the low dose was more effective. Investigators recently explained that the high dose of resveratrol could be ulcerogenic and disturb the ulcer-healing mechanism(s). In addition, a similar study proved that the low dose of resveratrol was effective in healing ulcers by inhibiting neutrophil aggregation and stimulating COX1, E2, and eNOS [53]. Similarly, our results showed that the treatment with high doses of resveratrol did not show any obvious improvement in gastric mucosal injury, which may be due to the high dose disturbing the healing mechanism(s). Collectively, we demonstrated that restraint stress injures gastric stomach parietal cells, which could lead to gastritis or ulceration, while the low dose of resveratrol treatment (2 mg/kg) protected the stomach from these untoward effects.

The dysfunction of parietal cells or their loss leads to increased proliferation of gastric mucosa cells, which can lead to an increased cellular apoptosis rate [54,55]. In this study, we performed IHC using an AIF antibody, and AIF is known to be one of the biomarkers for apoptosis. One study suggested that the increase in AIF expression in gastric epithelial cells can be an indicator of gastric tumors and a marker of parietal cell dysfunction [56]. Another study reported an increase in apoptosis in gastric mucosa with stress-induced gastric ulcers [57], which is similar to our present data as compared to the control group. Conversely, in some studies, resveratrol reduced apoptotic cell death caused by stress and other diseases [58,59,60,61]. Moreover, resveratrol inhibited inflammation through ostensibly independent effects on NF-κB, cyclooxygenase, interleukin-1β, inflammatory COX enzymes, STAT3, and HMGB1 [62,63,64,65]. In addition, mitochondria can be prevented from releasing AIF by down-regulating pro-apoptotic Bcl-2-associated X protein (BAX) which is up-regulated by anti-apoptotic proteins SIRT1, thereby suppressing BAX translocation into the mitochondria, and inhibiting the intracellular increase of Ca2+ caused loss of mitochondrial membrane potential. This is a crucial step in mediating intrinsic apoptotic cell death [66,67]. Our results showed a high expression of AIF in the cytoplasm and nuclei of parietal cells, and we demonstrated that restraint stress augmented apoptosis and dysfunction of parietal cells, while resveratrol attenuated the apoptotic rate and improved overall health.

Enlargement of the spleen (splenomegaly) is indicative of a vast range of diseases and can be detected by clinicians in many medical disciplines. This is because splenomegaly primarily appears as a secondary symptom in many diseases [68]. Some investigators using the same type of induced stress have reported a decrease in splenic weight [69,70,71]. Recently, one study on chronic stress showed splenomegaly with increased splenic weight [72]. Also, splenomegaly or splenic enlargement was found in other investigations [73,74,75], and one study depicting a significant role for oxidative stress in splenomegaly [76]. Furthermore, the enlargement of red and white pulp elements accompanied by the loss of the atypical structure of the germinal center (GC) leads to splenomegaly [77]. In animal models, splenomegaly can also arise as a physiologic response to stressors, and the increase in spleen size was associated with splenic extramedullary hematopoiesis (EMH) [16]. Additionally, disruption of the normal splenic architecture by a diffuse infiltrate predominantly expanded the white pulp. This infiltrate, when characterized by a proliferation of large atypical lymphoid cells with bizarre nuclei and prominent eosinophilic nucleoli surrounded by abundant cytoplasm, can lead to splenomegaly [78]. Our current study showed that restraint stress affected the size, length, and weight of the spleen. Furthermore, the white and red pulp was expanded with increased extramedullary hematopoiesis with abundant cytoplasms compared to the control group, and we demonstrated that resveratrol treatment protected the spleen and improved overall health. Accordingly, we recommend conducting more research on the relation between stomach disorders and splenic dysfunction in the case of resveratrol-treated restraint stress. However, further basic studies are also still needed with respect to splenic function and restraint stress. 

## 5. Conclusions

Restraint stress creates adverse effects on animal body weight, feeding, and water consumption. It also enhances the apoptotic process in parietal cells, which leads to the loss of cell number and size compared to the control group. Our findings showed that 2 mg/kg of resveratrol protected against the negative effects of restraint stress, which improved the health status compared with the control group. In addition, 2 mg/kg of resveratrol positively affected gastritis and splenomegaly caused by restraint stress; along with improved growth performance, food consumption, water consumption, and physiologic changes. In conclusion, 2 mg/kg of resveratrol reversed untoward effects on the stomach and spleen induced by restraint stress.

## Figures and Tables

**Table 1:** Relative levels of immunostaining for AIF antibody in the nucleus and cytoplasm of parietal cells.

Experimental Groups	Nucleus ^a^	Cytoplasm ^a^
Control	−	−
RS	+++	+++
RS + V	+++	+++
RS + 2mg RES	+	+
RS + 20mg RES	++	++
NC	N/A	N/A

^a^ Staining intensity: no staining detected (−); weak (+); moderate (++); strong staining (+++); N/A (not available).

## Supplementary Materials

The following are available online at https://www.mdpi.com/2076-2615/9/10/736/s1, Figure S1: The supplementary figure showed the brief experimental design including the restraint stress protocol for 4 h daily for 15 consecutive days.
