# Supplementary material for: Resveratrol Protects against Restraint Stress Effects on Stomach and Spleen in Adult Male Mice
Source: Animals (Basel). 2019 Sep 27;9(10):736. doi: 10.3390/ani9100736 (PMC6826970; doi:10.3390/ani9100736)
Supplement: Supplementary file 1 [file animals-09-00736-s001.pdf]

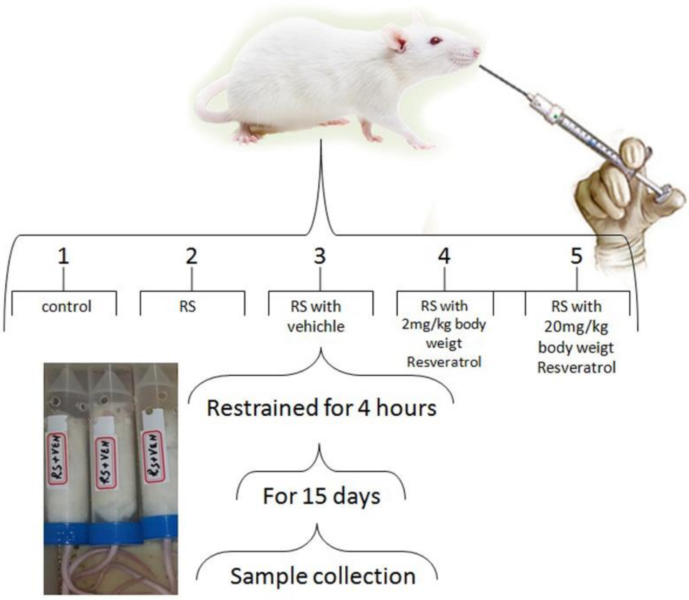

Figure 1. The supplementary figure showed the brief experimental design including the restraint stress protocol for 4 hours daily for 15 consecutive days.
